# COVID-19 and *Pasteurella multocida* Pulmonary Coinfection: A Case Series

**DOI:** 10.3390/tropicalmed7120429

**Published:** 2022-12-11

**Authors:** Ornella Cabras, Jean-Marie Turmel, Claude Olive, Bastien Bigeard, Mélanie Lehoux, Sandrine Pierre-Francois, Karine Guitteaud, Sylvie Abel, Lise Cuzin, André Cabié

**Affiliations:** 1 Infectious Diseases Department, University Hospital of Martinique, 97200 Fort-de-France, France; 2 Pathogenesis and Controle of Chronic and Emerging Infections, Univ Montpellier, Univ Antilles, INSERM, EFS, 34000 Montpellier, France; 3 Bacteriology Department, University Hospital of Martinique, 97200 Fort-de-France, France; 4 CERPOP, Université de Toulouse, Inserm UMR1295, UPS, 31000 Toulouse, France; 5 Center of Clinical Investigation of Antilles Guyane, INSERM 1424, 97200 Fort-de-France, France

**Keywords:** COVID-19 coinfection, *Pasteurella multocida* pulmonary infection, ventilator-acquired pneumonia

## Abstract

Objectives: In COVID-19 patients, bacterial and fungal pulmonary coinfections, such as *Streptococcus pneumoniae*, *Staphylococcus aureus*, *Haemophilus influenzae*, or *Aspergillus,* have been reported, but to our knowledge, no case has been reported due to *Pasteurella multocida*. Patients and methods: We describe three cases of *Pasteurella multocida* coinfections occurring during the 4th wave of COVID-19 in Martinique (French West Indies). Results: All three cases were fatal; thus, *Pasteurella multocida* has to be considered as a potentially severe coinfection agent. Conclusions: Alteration of the epithelial–endothelial barrier due to a SARS-CoV-2 infection probably promotes the expression of a *Pasteurella* infection. In addition, the SARS-CoV-2 infection induced immunosuppression, and an inflammatory cascade could explain the infection’s severity. The use of corticosteroids, which are part of the first-line therapeutic arsenal against COVID-19, may also promote the pathogenicity of this agent.

## 1. Introduction

At the end of 2019, the first cases of coronavirus disease 19 (COVID-19) were described in Wuhan, China, and the disease rapidly spread worldwide. On 27 October 2022, a total of nearly 630 million people had been infected by SARS-CoV-2. The first cases in Martinique were detected in March 2020. Since then, several waves happened in the French West Indies.

In addition, coinfection can occur during COVID-19. *Aspergillus fumigatus* or bacterial coinfections with *Streptococcus pneumoniae*, *Staphylococcus aureus,* or *Haemophilus influenzae* have been described in patients requiring mechanical ventilation for COVID-19 [1], but to our knowledge, no case has been described due to *Pasteurella multocida*.

In the meta-analysis of Bradley et al., a coinfection was reported in 3.5% (95%CI: 0.4–6.7%) of patients, and a secondary infection was reported in 14.3% (95%CI: 9.6–18.9%) of patients with COVID-19 [1].

*Pasteurella multocida* is a zoonotic pathogen with a multitude of hosts and is responsible for significant morbidity and mortality in both humans and animals [2]. *P. multocida* isolates can be classified into one of three subspecies, namely *multocida, septica*, or *gallicida.* In humans, the most frequent infections are local wound infections resulting from animal bites and scratches. The respiratory tract is the second most common site of *P. multocida* infections. Pulmonary infections seem to be more frequent in Martinique, where the breeding of farm animals is very common.

Here, we describe three cases of *P. multocida* coinfections among COVID-19 patients.

## 2. Detailed Case Descriptions

We collected oral consent from the patient families to use anonymous data.

No history of bites nor scratches was reported for any of the patients. None were vaccinated against COVID-19.

**Patient 1** was a 65-year-old, overweight (body mass index, BMI 29) man and farmer, with various farm animals and dogs at home (pigs, cattle, sheep, and goats). Cough, asthenia, anorexia, anosmia, and dyspnea appeared on 25 July 2021 (Day (D) 1). (Table 1).

On D10, he was admitted for acute respiratory failure with polypnea at 24/minute (min). Oxygen blood saturation (sAO_2_) was at 91% while receiving 15 L/min of oxygen (high concentration mask). Temperature was 37.2 °C.

A computed tomography scan (CT scan) showed an extensive interstitial syndrome, with bilateral ground glass opacities and crazy-paving lesions, and the HRCT score was severe [3]. Condensation areas were visible in the lower right and upper left lobes.

A blood test showed 12,900 leukocytes per mm^3^ and a C-reactive protein of 146 mg/L, normal transaminases, and normal renal function. Dexamethasone (6 mg per day) was started. The patient was transferred to the intensive care unit on D11 because of acute respiratory failure. Amoxicillin–clavulanic acid was started for a putative bacterial coinfection. Despite high-flow oxygen therapy, the patient worsened and required orotracheal intubation for mechanical ventilation.

On D14, endotracheal aspiration was positive with *P. multocida*. Blood cultures performed between D10 and D16 were sterile. Amoxicillin–clavulanic acid was continued pending the antibiogram. An increase in the blood leukocytes count led staff to perform a new endotracheal aspiration on D18, which again showed *P. multocida* with preserved susceptibility to penicillin; thus, the antimicrobial therapy was not modified. The new endotracheal aspirations, performed on D20 and D24, were negative.

On D25, sepsis worsened with hemodynamic and respiratory failure. An endotracheal aspiration found *Pseudomonas aeruginosa* and *Enterobacter aerogenes*; piperacillin and tazobactam were started. Despite this, he evolved to a refractory septic shock with major hypoxemia and died on D39. (Table 2).

**Patient 2** was an obese, (BMI 34) 72-year-old man with a history of hypertension. He was a former agricultural machinery driver. He had various animals at home (dogs, cats, pigs, and hens). (Table 1).

Fatigue and anorexia appeared on 29 August 2021 (D1). He was admitted on D7 due to dizziness and dyspnea. At admission, sAO_2_ was 84% without oxygen supply, and the respiratory rate was 35/min. He complained of anterior thoracic pain. His temperature was 36.1 °C, blood pressure 161/85 mmHg, and heart rate 75/min. A blood test showed a C-reactive protein of 119 mg/L, but a normal level of leukocytes and normal renal function. Ultrasensitive troponin was normal at 11.5 pg/mL. A CT scan showed bilateral ground-glass opacities, crazy-paving associated with bilateral base consolidations but no pulmonary embolism, and the HRCT score was mild [3]. He was started on O_2_ (6 L/min) and dexamethasone (6 mg per day), without antimicrobial therapy.

On D8, acute respiratory failure led to tracheal intubation for mechanical ventilation, and protected distal aspiration was then performed. On D9, he presented with hemodynamic failure and fever. Piperacillin–tazobactam, vancomycin, and amikacin were started. Noradrenalin was introduced after fluid loading without a hemodynamic response. The protected distal aspiration, performed on D8, was positive with *P. multocida*, 10^4^ CFU/mL. Blood cultures performed on D8 and D9 were positive with *P. multocida* and *S. aureus*. *P. multocida* was penicillin-susceptible, and *S. aureus* was methicillin-susceptible. The patient died on D10 of multiorgan failure and disseminated intravascular coagulation. (Table 2).

**Patient 3** was a 91-year-old man with a medical history of dilated cardiomyopathy, severe undernutrition, and esophageal stenosis. He had a dog, and he was a former butcher. (Table 1).

Fever, fatigue, and dyspnea appeared on September, 5th 2021 (D1). He was admitted on D11 because of increasing dyspnea. At admission, the physical examination showed sAO_2_ at 93% in ambient air, and his respiratory rate was at 28/min. He presented a general deteriorating condition. A blood test showed 9500 leukocytes per mm^3^, C-reactive protein at 160 mg/L, creatinine at 116 micromol/L, and elevated B-type natriuretic peptide (BNP) at 2203 pg/mL. Arterial blood gas with ambient air found a pH of 7.50, pO_2_ 49 mmHg, pCO_2_ 29 mmHg, and bicarbonate 25 mmol/L. A CT scan showed ground-glass opacities with crazy-paving reaching 25 percent of the parenchyma, a small condensation in the lower left lobe, and no pulmonary embolism, and the HRCT score was mild [3]. Oxygen 10 L/min with a mask and dexamethasone (6 mg per day) were started at D12.

On D14, the oxygen requirement increased to 15 L/min. On D15, fever appeared, and the C-reactive protein was at 256 mg/L. On D13, the blood culture was positive with *P. multocida.* No sputum could be analyzed. Amoxicillin–clavulanic acid was started. D16 and D17′s blood cultures were sterile, but the D18 blood culture was again positive with *P. aeruginosa,* and inflammatory markers improved.

Because of the patient’s condition and his wish to refuse extensive care, he was not admitted to the intensive care unit and died on D21. (Table 2).

## 3. Discussion

*Pasteurella multocida* is a Gram-negative coccobacillus able to colonize animals’ oral cavities and gastrointestinal tracts leading to skin and soft tissue infections after animal bites. Human infection can also occur via the inhalation of contaminated secretions [2]. Professional animal contact (veterinarians, butchers, animal breeders, and farm and zoo workers) and pet ownership increase the possibility of subclinical carriage or infection [4].

The respiratory tract is the second most common site of *Pasteurella* infections, with possible pneumonia, tracheobronchitis, lung abscess, or empyema. Patients with a *Pasteurella* pulmonary infection are often elderly and have an underlying lung disease, either chronic obstructive pulmonary disease (COPD), bronchiectasis, or malignancy [5,6,7,8,9,10,11,12].

A variety of other serious invasive infections, such as meningitis, bacteremia, endocarditis, and peritonitis, have also been reported, but are rare. Bacteremia occurs preferably in immunocompromised patients.

Some cases of acute pneumonia due to *P. multocida* have been reported [6,7,8,9,11,12], but no cases have been reported as a COVID-19 coinfection. The three cases reported here occurred during the same epidemic wave and ended with death in all cases, and thus are a warning to consider *P. multocida* as a potentially severe coinfection agent. Of note is that two out of the three cases had other bacterial-associated infections, namely *P. aeruginosa* or *S. aureus*, which were probably in part responsible for the severity of the clinical presentations. No history of bites or scratches was documented in our patients, but the presence of animals, potentially carriers of *Pasteurella*, was found in each of them. None of these patients received monoclonal antibodies, tocilizumab, or any anti-IL6 factor, but they all received dexamethasone.

It has been shown in animals that infection with a respiratory virus can lay the foundation for a *Pasteurella* infection [2,13]. Environmental conditions and the animal’s condition can influence the severity of the disease. The *Pasteurella* species produces several virulence factors. The constitution of lipopolysaccharides associated with a hydrophilic capsule allows to escape the immune system. Several outer membrane proteins serve as an adhesin by binding to its host. Finally, the bacteria produce toxins. All these virulence factors are expressed according to the serotypes [13]. Unfortunately, serotyping was not available for the three strains of our patients.

Because of the short delay between the beginning of the symptoms and positive samples of *P. multocida* (less than 7 days for all cases), we classified the cases as community-onset infections. We hypothesized that the patients were contaminated by close contact with their animals. A SARS CoV-2 infection probably promoted the expression of the *Pasteurella* infection by alteration of the epithelial–endothelial barrier. In addition, the SARS-CoV-2-induced inflammatory cascade leading to immunosuppression could explain the infection’s virulence. The use of dexamethasone, which is part of the first-line therapeutic arsenal against COVID-19, may also facilitate the development of this coinfection.

We know that incidences of *P. multocida* are more frequent on Martinique due to the living conditions and close contact with many animals and could, for this reason, increase at the same time the incidence rate of SARS-CoV-2 infections.

*Pasteurella multocida* does not have any difficulty in growing on bacteriological media. It grows well on blood agar and chocolate agar. However, it can be difficult to isolate due to overgrown commensal flora on the agar. It may be misidentified due to the phenotypic resemblance it may have to *Haemophilus* or *Neisseria*.

## 4. Conclusions

Here we report three cases of *P. multocida* coinfections among COVID-19 patients, all of which evolved into death.

A *Pasteurella multocida* coinfection with SARS-CoV-2 seems to be of serious concern and needs to be considered by the persons taking care of these patients.

## Figures and Tables

**Table 1:** Patients’ characteristics at admission.

	Sex/Age	Medical History	Potential Exposure	Temperature (°C)	S_a_O_2_ *	Respiratory Rate (/mn)	CRP * (mg/L)
**Patient 1**	Male/65	BMI * = 29	Various farm animals Dogs	37.2	91 O_2_ 15 L/min	24	146
**Patient 2**	Male/72	BMI * = 34	Various farm animals Dogs, cats	36.1	84 Ambient air	35	119
**Patient 3**	Male/91	Dilated cardiomyopathy Undernutrition Esophageal stenosis	Dog	37.5	93 Ambient air	28	160

* S_a_O_2_: *oxygen saturation*; CRP: *C-Reactiv Protein*.

**Table 2:** Disease evolution.

	Bacteriology Assessments	Symptom Onset	Coinfection	Antimicrobials	Outcome
**Patient 1**	Endotracheal aspiration D14 and D18	D14	SARS-CoV2 P. aeruginosa * E. aerogenes *	Amoxicillin–clavulanic acid	Death D39
**Patient 2**	Protected distal aspiration D8 Blood culture D8 and D9	D8	SARS-CoV2 S. aureus *	Piperacillin–tazobactam Vancomycin Amikacin	Death D10
**Patient 3**	Blood culture D13	D13	P. aeruginosa *	Amoxicillin–clavulanic acid followed by Amoxicillin	Death D21

* *P. aeruginosa*: *Pseudomonas aeruginosa*; *E. aerogenes*: *Enterobacter aerogenes*.

## Data Availability

Not applicable.
